# Bochun: Automatically annotated stance detection dataset for Sorani Kurdish language

**DOI:** 10.1016/j.dib.2025.111839

**Published:** 2025-06-25

**Authors:** Payman Sabr Rostam, Rebwar Mala Nabi

**Affiliations:** a Information Technology Department, Technical College of Informatics, Sulaimani Polytechnic University, Sulaimani, Kurdistan Region, Iraq; b Information Technology Department, Kurdistan Technical Institute, Sulaimani, Kurdistan Region, Iraq

**Keywords:** Stance detection, Automated labelling, Low-resource languages, NLP, Pattern recognition, Kurdish Sorani language, Zero-shot learning

## Abstract

This Research presents the first-ever, high-quality, automatically annotated Kurdish stance detection dataset in the Sorani dialect to fill the gap of lacking annotated resources for Kurdish, a low-resource language in Natural Language Processing (NLP). The dataset consists of 2,174 Kurdish news articles—1,410 economic and 764 political—that were originally published in 2024 and 2025, which are recent and topically relevant. By selecting these texts from well-known Kurdish news agencies, content validity and linguistic purity were preserved throughout. Necessary preprocessing techniques are applied. Annotation is carried out in two steps. First, a pattern-recognition method with 2,456 phrases and keywords was applied to determine if the subject of every text fell into the economics or politics category. Next, the position of every article was annotated with an extended lexicon of 4,243 adjectives and verbs, categorized under support, oppose, and neutral. Wherever direct matches were not possible, semantic similarity and zero-shot classification were used as fallback measures. In order to verify the automatic annotation, a team of domain experts manually assessed a representative sample of the annotated texts, with a high inter-annotator agreement score confirming the validity of the approach. The dataset is made available in XLSX (Excel) format, facilitating ease of use and versatility for a variety of research tasks in NLP. Due to its annotated and organized corpus, this dataset is a solid starting point for researchers who are building Kurdish language processing models. The dataset is released publicly to allow other researchers to build upon it and push the limits of NLP system performance on low-resource languages.

Specifications Table

**Table:** 

Subject	Computer Sciences
Specific subject area	Natural Language Processing, stance detection in Sorani Kurdish language
Type of data	Microsoft Excel format .xlsx
Data collection	Here, we tackle stance detection in 2024 and beyond Kurdish news articles. We automatically extracted the data from open Kurdish news websites using Python scripts, then cleaned and normalized it. We put the resulting texts into Excel files, where a pattern-recognition approach was employed both for annotating the data as well as for identifying each article's target and stance. The data is finally stored in XLSX (Excel) format, for easy consumption into the majority of NLP pipelines.
Data source location	https://www.rudaw.net/sorani/business https://www.rudaw.net/sorani/kurdistan https://www.rudaw.net/sorani/middleeast/iraq
Data accessibility	Repository Name: Mendeley Data • Data identification number: 10.17632/ckkxx8mdcg.5 • Direct URL to data: https://data.mendeley.com/datasets/ckkxx8mdcg/5 • Instructions for accessing the data: Publicly available for research purposes
Related research article	None

## 1 Value of The Data

• The dataset is a cornerstone digital resource for Sorani Kurdish as a low-resource language in NLP. It mitigates the lack of annotated datasets by providing a strong basis for research and development in Kurdish language processing. Because of its multi-domain coverage and versatility, the dataset can be reprocessed for a wide range of NLP tasks, such as stance detection, Named Entity Recognition (NER), sentiment analysis, machine translation, and text classification. This flexibility guarantees widespread application across various research agendas and practical applications, advancing Kurdish NLP as a field.
• Annotated by a pattern-recognition-based system, the dataset is of high consistency and accuracy in stance information labeling. The method takes advantage of a systematically developed lexicon of stance keywords, along with automatic text preprocessing for content cleaning and normalization as a prerequisite for annotation. Where needed, the annotations are verified and enriched by domain experts for reliability. Such a rigorous annotation process not only serves stance detection research but also provides a replicable system for other low-resource language research.
• The dataset serves as a benchmark for the assessment of Kurdish stance detection models so that researchers can quantify system performance and compare results between approaches. Because it includes articles from various domains—e.g., politics and economics—it requires models to generalize well to a range of linguistic contexts.
• The data is made available in XLSX (Excel) format to be usable in most data processing pipelines. As it is openly accessible, it promotes collaboration since researchers can replicate experiments, extend new models, or perform cross-linguistic comparisons. With open documentation of collection and annotation practices, the data also allows for reproducibility and methodological improvement.

## 2 Background

Kurdish is an Indo-Iranian language spoken by an estimated 30 to 40 million people across Turkey, Iraq, Iran, Syria, and Armenia, making it one of the most widely spoken stateless languages in the world. These dialects vary not just linguistically but also in their writing systems: Kurmanji is mostly written in the Latin script, while Sorani employs an Arabic-based script, which complicates Natural Language Processing (NLP) activities and standardization. Usually, Kurdish has a Subject-Object-Verb (SOV) syntactic structure and exhibits characteristics like ergativity in the past tense and rich morphology. Kurdish is still very underrepresented in computer linguistics given its great use because of insufficient standardized tools and low technological investment [1,2].

The dialect used in this study, Sorani Kurdish, is officially recognized in the Kurdistan Region of Iraq and is extensively used in government, education, and media [3]. In NLP, though, it is still a low-resource language with no previously published stance detection datasets accessible. This paper presents the first annotated stance detection dataset for Sorani Kurdish.

A subfield of NLP called stance detection classifies a text's position toward a particular target as support, oppose, or neutral [4]. Although this work has been extensively researched in high-resource languages like English and Arabic, the Sorani dialect of Kurdish has had no annotated materials for stance detection. Being a low-resource language, Sorani Kurdish struggles more with NLP because of its particular script, small labeled corpora, and absence of uniform tools [5]. Our dataset—made up of news articles from 2024 to 2025 in the fields of politics and economics—aims to support reproducibility and allow downstream NLP research for Kurdish and other underrepresented languages, therefore addressing this gap.

This work greatly adds to the expanding collection of Kurdish NLP resources by aiming Sorani Kurdish and building a stance-labeled dataset, so laying the groundwork for next tools and models.

## 3 Data Description

The Kurdish Stance Dataset is organized into a main folder called Kurdish_Stance_Dataset, which has two subfolders: data (for files) and code (for scripts). Below is a description of the main files in the data folder:

• Bochun_Orginal_Data.xlsx
  This is the original dataset of news articles collected from the Rudaw website. Each row is one article, including the title, full text, publish date, link, category, and date it was accessed.
• Target_Lexicon.xlsx
  A list of 2,456 keywords and phrases related to economy and politics. These were used to help identify the main topic of each article.
• Bochun_Target.xlsx
  This file is the result of applying our target identification process to the original articles using the target lexicon. It includes all the original columns plus a new one called target, which shows the main topic detected for each article (like “currency”, “budget”, or “election”).
• Stance_Lexicon.xlsx
  A list of 4,243 stance-related words and phrases. They are divided into three categories: support, oppose, and neutral. These help determine the writer’s position in each article.
• Bochun_Stance_Kurdish.xlsx
  This is the final version of the dataset. It adds a stance column to Bochun_Target.xlsx, showing whether the article supports, opposes, or stays neutral on the topic.
• readme.md
  A guide that explains the dataset structure, what each file contains, and how to use the data.

All Python scripts that process these files are located in the code folder.

**Fig. 1:**
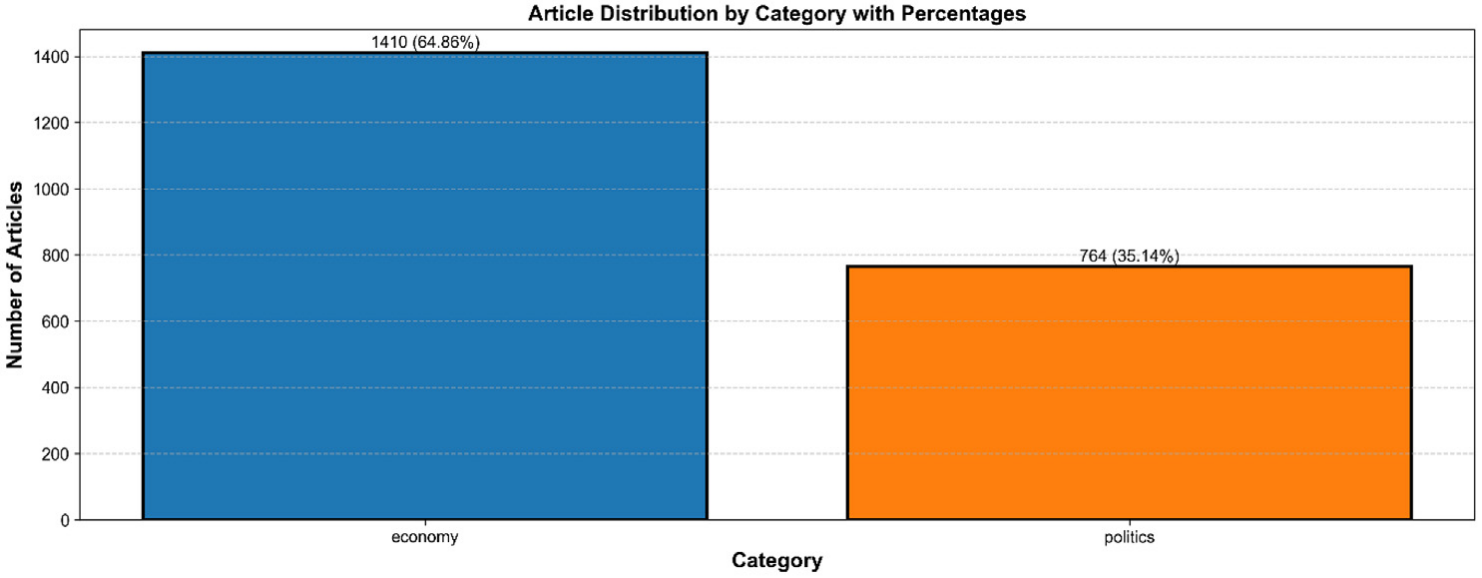
Article distribution by category.

In Fig. 1, the distribution of news articls in the “Economy” and “Politics” categories is illustrated. As is evident from the chart, there are greater stories under the “Economy” category, indicating greater media attention or coverage of economic affairs during the chosen time frame.

**Fig. 2:**
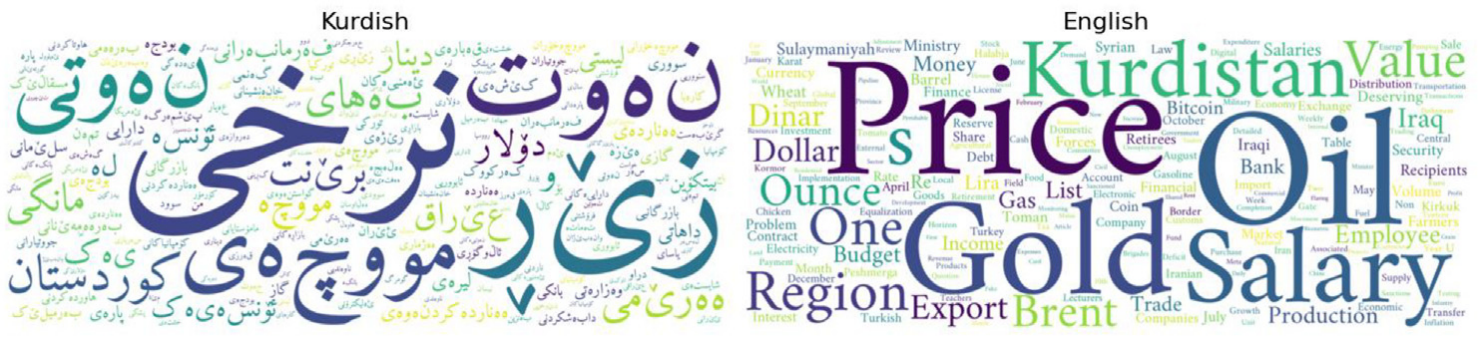
Visualization of the most frequent economic keywords.

Fig. 2 shows the number of economic keywords in the dataset. It represents the most frequent economic keywords, providing a glimpse of significant economic themes and issues reported in the news stories.

**Fig. 3:**
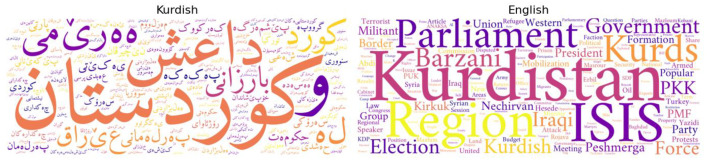
Visualization of the most frequent political keywords.

Fig. 3 shows the frequency of political keywords in the dataset. It represents the most frequent political keywords, identifying significant political themes and controversies in the news stories.

**Fig. 4:**
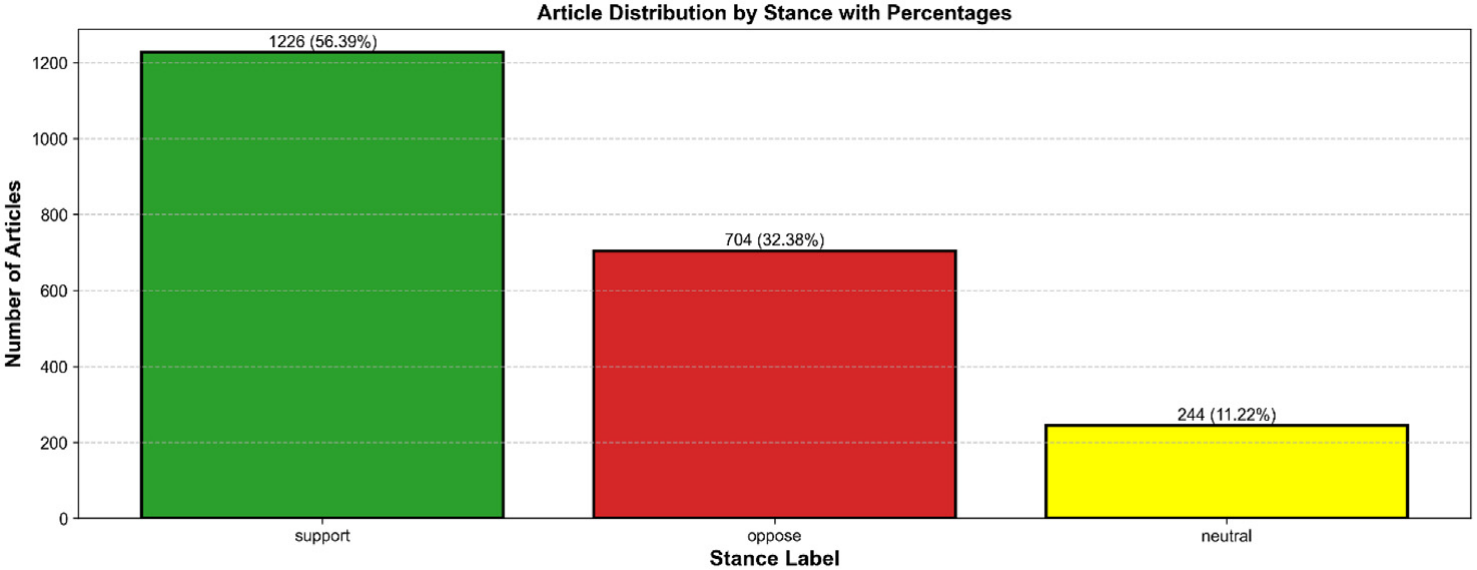
Article distribution by stance labels.

Fig. 4, demonstrate the breakdown of the labels of stance—support, oppose, and neutral—across the dataset shows which of the stances are most prevalent, helping to identify bias and to convey overall sentiment of the media coverage.

## 4 Experimental Design, Materials and Methods

### 4.1 Data Collection and Categorization

We collected news articles from the Kurdish news portal Rudaw (http://www.rudaw.net) between March 2024 and February 2025. We crawled three thematic sections of the website with category-specific URLs:

• Business (1,562 articles)
• Kurdistan (1,988 articles)
• Middle East/Iraq (1,000 articles)

This initial scraping resulted in a total of 4,550 articles. Considering that Rudaw is one of the most professional and reputable news agencies in the Kurdistan Region of Iraq—and that it releases news articles in the Sorani Kurdish dialect—we selected it as the single provider for this dataset to maintain linguistic consistency and content reliability.

Since the predefined news categories on the Rudaw website do not entirely align with the exact domains of our desired dataset—i.e., Economy and Politics—we conducted a manual review and filtering of the scraped articles. Irrelevant articles were removed according to their content, resulting in a final curated dataset of 2,174 relevant articles, each uniquely associated with either the economy or politics domain.The process for assigning domains was as follows:

✓ Articles selected from the Business section were examined and kept in the Economy category.
✓ All Middle East/Iraq and Kurdistan section news articles were screened manually, and only articles specific to governance, policy, political events, or public administration were retained under the Politics category.

The last dataset distribution is:

• Economy: 1,410 articles from 1,562 initially scraped Business articles with retention rate: approximately 90.3 %.
• Politics: 764 articles from 2,988 articles collected from Kurdistan and Middle East/Iraq categories with retention rate: approximately 25.6 %.

This refers to the lack of quality political content in contrast to the availability of data collected and recognizes the difficulty of accessing low-resource languages with skewed category systems. To enable the automation of data scraping, a two-stage pipeline built in Python was used. The first stage used a headless Selenium driver to render dynamic content (https://www.selenium.dev/) by performing actions like clicking the “Show all” button and performing a page scroll. Then, the HTML content fetched was parsed using BeautifulSoup to extract article URLs. In the second stage, each article page was visited individually using the requests library (https://requests.readthedocs.io/en/latest/) where structured elements like titles, publication dates, and body text were extracted by applyingBeautifulSoup (https://www.crummy.com/software/BeautifulSoup/). These tools enabled automated collection of thousands of articles from Rudaw with minimal human interaction, ensuring efficiency and consistency during the data acquisition process.

The Python script used for the automated scraping process (scraper.ipynb) is publicly available along with the dataset on Mendeley Data, enabling full reproducibility of the data collection procedure.

### 4.2 Preprocessing

As the scraped content of the chosen news portal was clean, there was minimal preprocessing in data collection. In annotation, though, certain text normalization was done for consistency. In particular, we used two general methods:

(1) Redundant Suffix Removal: Python scripts were employed to discover and eliminate redundant word suffixes via regular expressions and a precompiled list of typical Kurdish suffixes. For instance, an article title that had the word “” (where “-” is a redundant plural suffix) was normalized to “.”
(2) Removal of Non-Essential Introductory Text: Non-essential introductory text was removed manually as a precursor. For example, an article starting with:
  “.”

“Rudaw Digital. After the rise of Bashar al-Assad's regime, the price of fuel in Syria has continued to rise, and compared to the rise during the reign of Bashar al-Assad, it has increased by nearly 200 %.” was transformed into:

“.”

“After the rise of Bashar al-Assad's regime, the price of fuel in Syria has continued to rise, and compared to the rise during the reign of Bashar al-Assad, it has increased by nearly 200 %.”

These targeted adjustments ensured that the textual content was standardized for the subsequent pattern-recognition-based annotation process.

### 4.3 Annotation Methodology

**Fig. 5:**
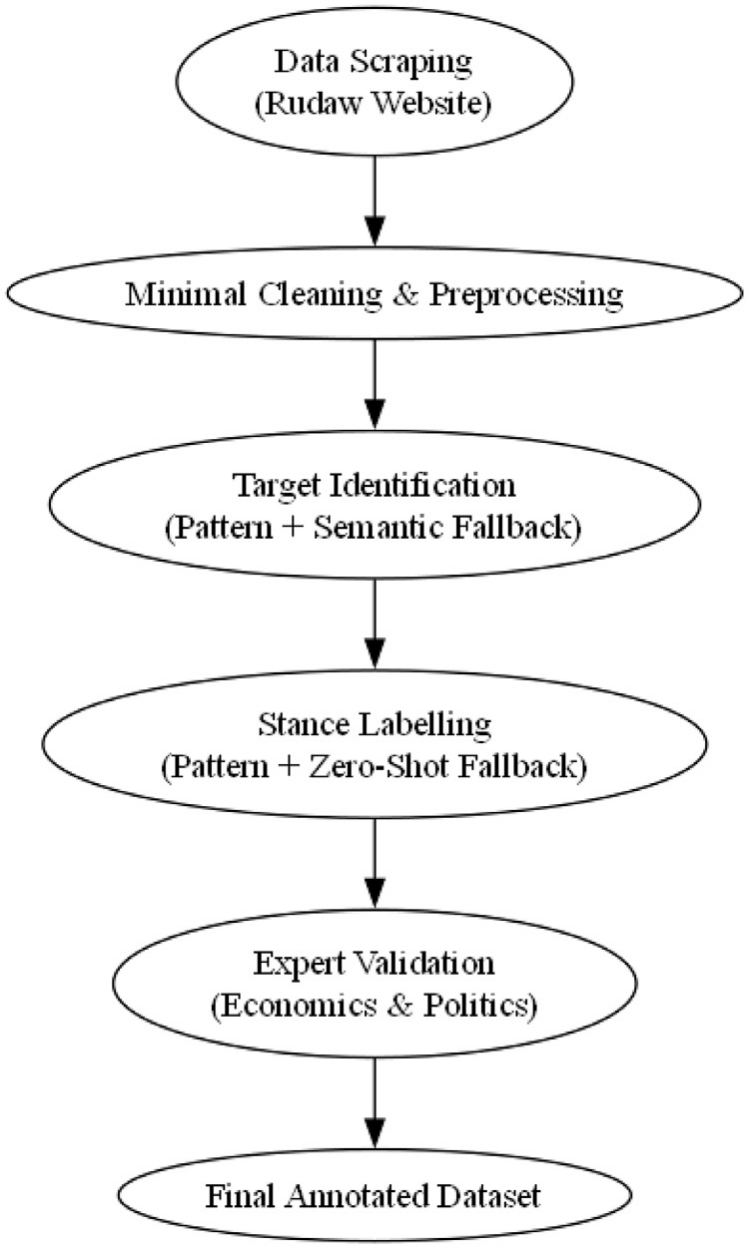
End-to-end pipeline for data collection and annotation. The process includes scraping articles from Rudaw, preprocessing, identifying targets, applying stance labels using a hybrid method, and inal expert validation to ensure dataset quality.

The flow of work for the study, starting from data scraping and preprocessing to target identification, stance labelling, and validation by experts, is laid out in the flowchart below in Fig. 5. This gives a step-by-step explanation of the transparent methods used and enables transparency and reproducibility in research.

#### 4.3.1 Target Identification

The initial dataset consists of 2,174 Sorani Kurdish news articles categorized under “Economy” and “Politics”. The data is provided in an Excel spreadsheet (Bochun_Orginal_Data.xlsx), with each row containing the article’s title, full content, publication date, web link, access date, and category. To determine the domain-specific target for each article (e.g., “currency” , “election” , “budget” ), we implemented a Python-based target identification pipeline.

This pipeline is built around a hybrid strategy that combines rule-based keyword matching with semantic similarity techniques. A domain-specific lexicon (Target_Lexicon.xlsx) containing 2,456 words and phrases relevant to economy and politics was used as a reference. The script first normalizes the article titles using the regex Python library to remove any non-Arabic characters except digits and spaces. This ensures compatibility with the Sorani script while preserving numerical references.

To improve lexical precision, the system dynamically fetches a list of common prefixes and suffixes from an external API (ghaziabdullah.pythonanywhere.com) using the requests library. These affixes are stripped from words only if doing so reveals a known keyword from the lexicon. This step enhances the detection of morphologically complex or inflected forms that are common in Kurdish.

The main identification process prioritizes exact matches for compound keywords (multi-word expressions) in the article title. If no match is found, the script processes the title on a word-by-word basis, applying the affix-handling logic and searching again for any known keyword. If no match is still found, the script falls back to a semantic similarity method using the sentence-transformers library. Specifically, it employs the multilingual model paraphrase-multilingual-mpnet-base-v2(sentence-transformers/paraphrase-multilingual-mpnet-base-v2) from Hugging Face, which supports over 50 languages including Kurdish. Each candidate phrase and the processed article title are converted into dense vector representations (embeddings), and the most similar target is identified using cosine similarity via the scikit-learn (https://scikit-learn.org/stable/getting_started.html) library.

The resulting target domain is then added as a new column in the output file (Bochun_Target.xlsx). After the automatic identification process, a manual review was conducted to validate and correct the assigned targets where necessary, ensuring a high level of accuracy in the final dataset.

The overall procedure is illustrated in Algorithm 1, which shows the pseudocode for our target identification pipeline The full implementation of the target identification script (target_identifier.ipynb), along with the annotated dataset, is publicly available on Mendeley Data

**Algorithm 1:** Outlines the pseudocode for our target identification pipeline.

Inputs: - bochun_original.xlsx - Lexicon file (2,457 words/phrases) - Online prefixes/suffixes list Output: - bochun_target.xlsx BEGIN articles ← LOAD_EXCEL("bochun_original.xlsx") lexicon ← SORT_BY_LENGTH_AND_WORDCOUNT(LOAD_LEXICON("lexicon_file.xlsx")) prefixes, suffixes ← GET_HTTP_AFFIXES() model ← LOAD_SENTENCE_TRANSFORMER("paraphrase-multilingual-mpnet-base-v2") lexicon_embeddings ← model.ENCODE(lexicon) FOR each article in articles DO title_clean ← RETAIN_ARABIC_DIGITS_SPACES(article.title) // Check for compound word match target_found ← FIND_TARGET_IN_TEXT(title_clean, lexicon, compound=True) IF target_found is NULL THEN tokens ← SPLIT_INTO_TOKENS(title_clean) processed ← [SAFE_STRIP(t, prefixes, suffixes) for t in tokens] text_processed ← JOIN(processed, “ ”) target_found ← FIND_TARGET_IN_TEXT(text_processed, lexicon, compound=False) ENDIF IF target_found is NULL THEN embedding ← model.ENCODE(text_processed) target_found ← lexicon[ARGMAX_COSINE_SIM(embedding, lexicon_embeddings)] ENDIF article.target_domain ← target_found ENDFOR SAVE_EXCEL(articles, “bochun_target.xlsx”) PERFORM_MANUAL_REVIEW("bochun_target.xlsx") END

#### 4.3.2 Stance Labelling

Stance annotation was accomplished through a bespoke Python pipeline, which labeled each article in a systematic manner as supportive, opposing, or neutral with respect to its target. Annotation was undertaken using a hybrid strategy combining lexicon-based pattern matching, semantic similarity assessment, and zero-shot classification techniques.

The pipeline utilized a manually curated stance lexicon of 4,241 Kurdish Sorani words and phrases, divided into three classes:

• Support: Positive or favorable tone toward the target
• Oppose: Negative or critical tone toward the target
• Neutral: Neither clearly positive nor negative

We followed these annotation guidelines during system development and human validation:

1. If the article included one or more stance-indicating words near the identified target, it was labeled accordingly.
2. If the tone was clearly favorable, terms such as “” (support), “” (best), or “” (beneficial) indicated support.
3. If the article included criticism or negative tone such as “” (bad), “” (decrease), it was labeled oppose.
4. If no opinionated language was found, or the tone was factual, the stance was marked as neutral.

In case a direct keyword match was not available, the system applied semantic similarity through Sentence Transformers and cosine similarity. In case ambiguity still existed, a zero-shot classifier (joeddav/xlm-roberta-large-xnli) from Hugging Face Transformers was applied with candidate labels (support, oppose, neutral).

The entire process was implemented in Python using open-source NLP libraries. Human validation by domain experts was also utilized to ensure that label quality was guaranteed (Section 4.3.3).

Algorithm 2 prescribes the pseudocode for our stance labelling pipeline, with each article being processed to detect a stance (support, oppose, or neutral) using a combination of proximity-based detection and, as necessary, a zero-shot classifier. The full implementation of the annotation script (stance_annotator.ipynb), along with the annotated dataset, is publicly available on Mendeley Data.

**Algorithm 2:** Stance labelling pipeline.

Inputs: - bochun_target.xlsx - stance_pattern.xlsx - Kurdish affixes list - Zero-shot classifier configuration Output: - bochun_stance.xlsx BEGIN 1. Load articles from bochun_target.xlsx into articles 2. Load stance keywords from stance_pattern.xlsx into stance_keywords 3. Define Kurdish affixes (prefixes and suffixes) 4. Initialize zero-shot classifier with model “joeddav/xlm-roberta-large-xnli” and candidate labels [“support”, “oppose”, “neutral”] 5. For each article in articles: 5.1 If title and content are empty, assign empty values for stance, target_variant, and stance_keyword; then continue to the next article 5.2 Clean and trim the target value from the article 5.3 Evaluate stance in the title: stance, target_variant, stance_keyword ← detect_stance_proximity_based(title, target, stance_keywords, affixes) If stance is found, assign it to the article and continue to the next article 5.4 If no stance is found in the title: Split content into sentences For each sentence in sentences: stance, target_variant, stance_keyword ← detect_stance_proximity_based(sentence, target, stance_keywords, affixes) If stance is found, assign it to the article and break the loop 5.5 If stance is still not found: Select text ← title if available, otherwise content result ← zero-shot classifier(text, candidate_labels, hypothesis template including target) stance ← top label from result 5.6 Append stance, target_variant, and stance_keyword to the article record 6. Save articles with new stance columns to bochun_stance.xlsx END

#### 4.3.3 Labelling Validation and Verification

As described in Section 4.3.2, our corpus was initially auto-annotated with our hybrid stance detection pipeline based on lexicon-based proximity detection, semantic similarity, and a zero-shot classifier. We then had four native Sorani Kurdish experts—two economics and two political journalism experts—manually annotate a random subset of 450 economy articles (31.9 % of 1,410) and 250 politics articles (32.7 % of 764).

After refining the annotation guidelines to match the specified review, the entire corpus of 2,174 articles was annotated manually by the same group of experts. All differences in the annotations were resolved through mutual discussion among the authors and the annotators. We then calculated Cohen's kappa from these precise manual labels, which yielded coefficients of 0.90 for the economic domain and 0.93 for the political domain.

The near-optimum concordance values confirm the reliability and validity of our hybrid automatic and manual annotation process.

This brief validation step guarantees the resulting stance-annotated corpus to be reproducible, consistent, and reliable for any future NLP study.

An overview of the finished stance-annotated dataset is shown in Table 1, which includes important columns like the article title, domain category, identified target, and assigned stance label. The examples show the dataset's structural arrangement as well as the useful results of the annotation process. They show how stance labels—support, oppose, or neutral—were assigned using a combination of automated and human-reviewed methods, and how articles were divided into economy and politics based on the content and source section. The table provides a succinct overview of the dataset's final structure and labeling quality, highlighting the articles' thematic diversity as well as the consistent application of annotation criteria.

**Table 1:** Sample of labeled data from the dataset.

## Limitations

One of the limitations of this dataset is its time frame, as it works with Kurdish news stories that were primarily published in 2024 and 2025 and do not necessarily contain historical or future trends in the topics they address. The dataset is also economics- and politics-oriented, which may not represent the full scope of societal issues and Sorani Kurdish linguistic dialects. This domain specificity could limit the potential use of the dataset for other more diverse NLP tasks with different subject matter.

## Ethics Statement

The authors declare that this work is in accordance with the ethical standards of Data in Brief publishing. The dataset was compiled from publicly available news articles, and no personal or sensitive data were used. There were no human subjects or animal experiments involved in this research.

## CRediT authorship contribution statement

**Payman Sabr Rostam:** Conceptualization, Data curation, Methodology, Software, Validation, Formal analysis, Writing – original draft, Visualization, Project administration, Funding acquisition. **Rebwar Mala Nabi:** Supervision, Writing – review & editing.

## Data Availability

(Mendeley Data).Kurdish dataset for stance detection (Original data)

## Appendix Supplementary materials

Image, application 1

Image, application 2
