# Supplementary material for: Bochun: Automatically annotated stance detection dataset for Sorani Kurdish language
Source: Data Brief. 2025 Jun 25;61:111839. doi: 10.1016/j.dib.2025.111839 (PMC12266528; doi:10.1016/j.dib.2025.111839)
Supplement: Supplementary file 1 [file mmc1.docx]

# This document includes all the figures, and diagrams referenced in the main manuscript. It provides a comprehensive visual representation of the data and methodologies discussed throughout the paper.


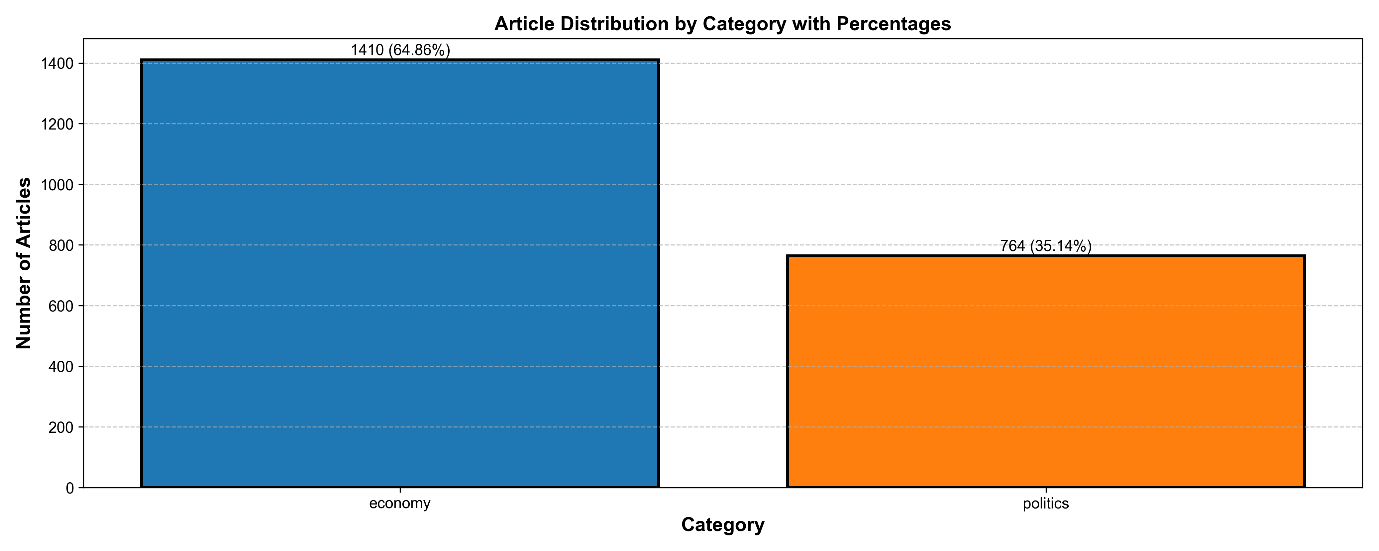


**Fig. 1.** Article Distribution by Category.


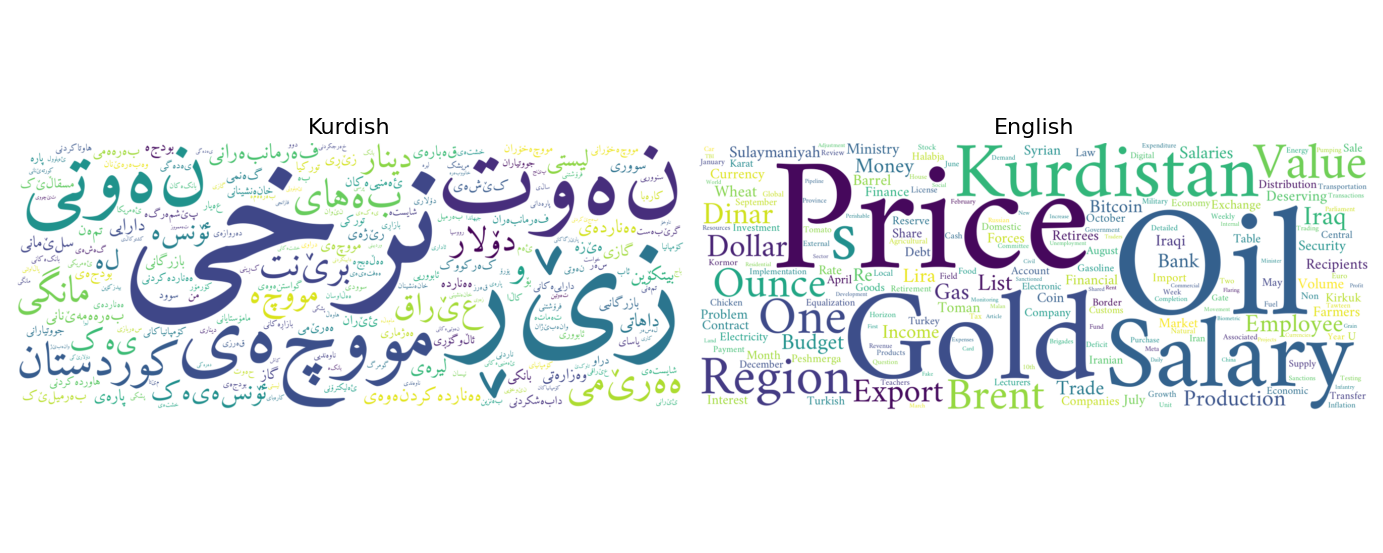
**Fig. 2**. Visualization of the Most Frequent Economic Keywords.
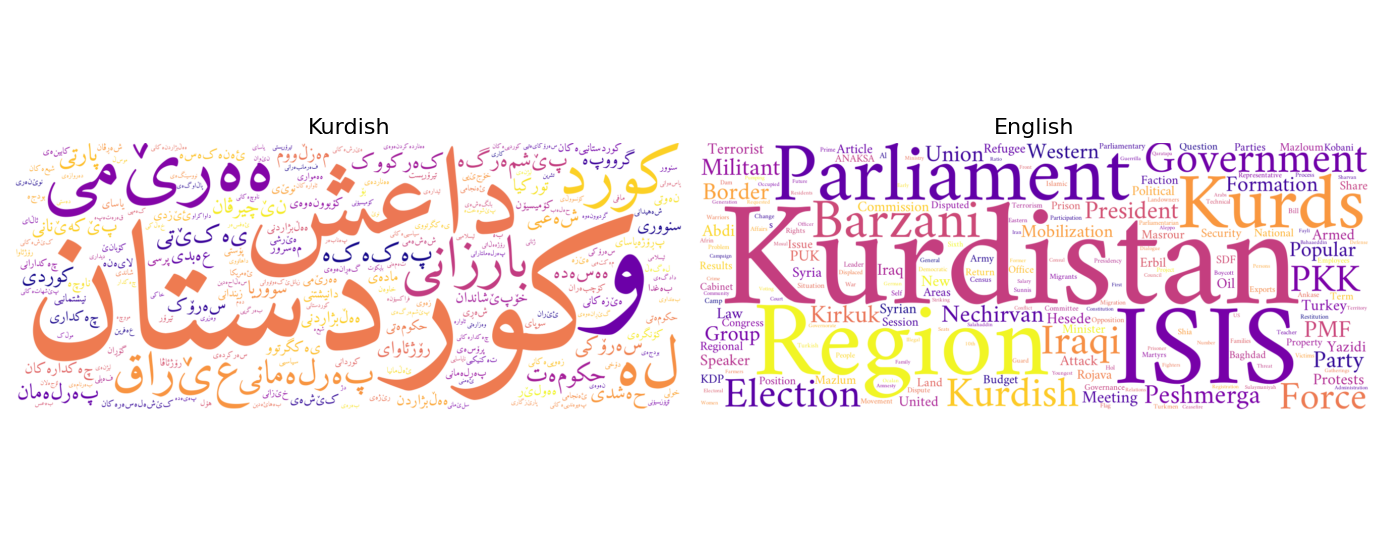


**Fig. 3.** Visualization of the Most Frequent Political Keywords


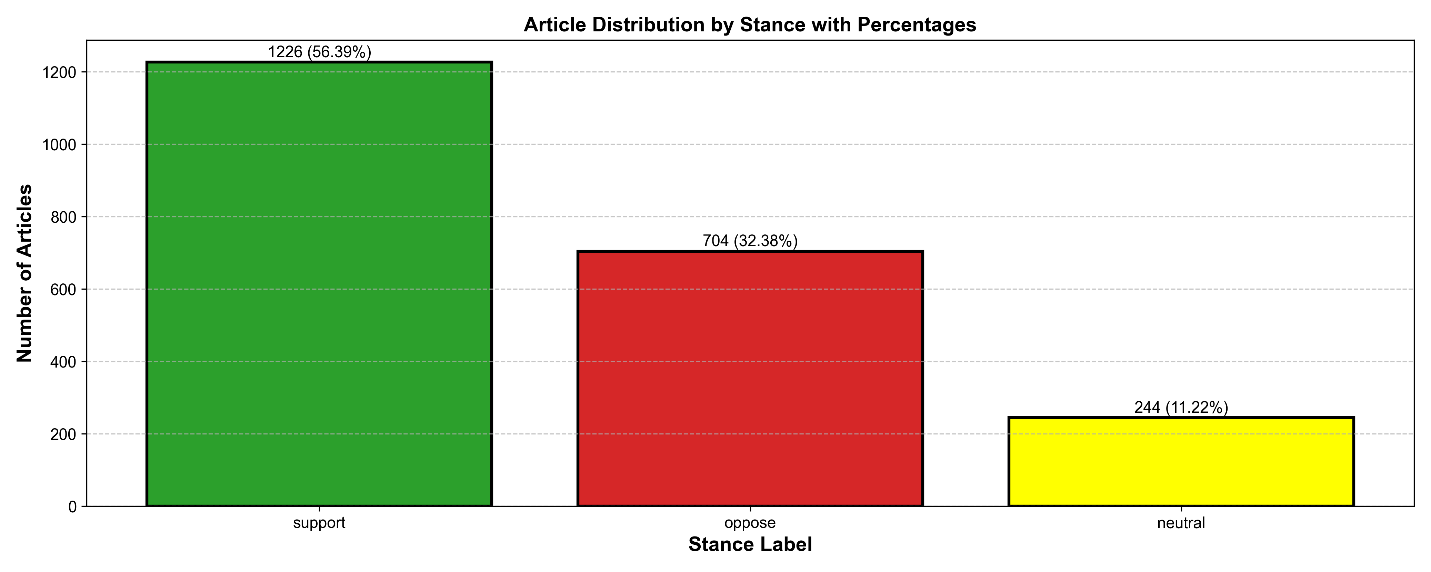


**Fig. 4.** Article Distribution by Stance Labels.


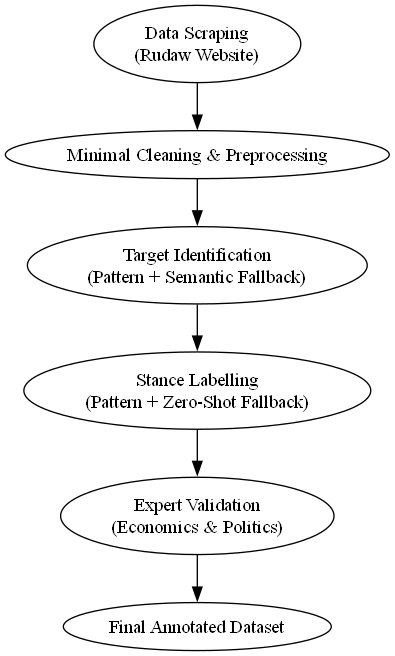


**Fig. 5.** Methodology of our data collection and labelling.
